# Community Genetic Services in Iran

**DOI:** 10.1155/2012/129575

**Published:** 2012-12-05

**Authors:** Shirin Atri Barzanjeh, Mozhgan Behshid, Mohammad Bagher Hosseini, Maryam Ezari, Mahdieh Taghizadeh, Saeed Dastgiri

**Affiliations:** ^1^School of Nursing and Midwifery, Tabriz University of Medical Sciences, Tabriz 5166615739, Iran; ^2^Health System Research Committee, Tabriz Health Services Management Research Centre, Tabriz University of Medical Sciences, Tabriz 5166615739, Iran; ^3^Pediatric Health Research Centre, Tabriz University of Medical Sciences, Tabriz 5166615739, Iran; ^4^Tabriz Health Services Management Research Centre, School of Medicine, Tabriz University of Medical Sciences, Tabriz 5166615739, Iran

## Abstract

The aim of the study was to report a description of the primary, secondary, and tertiary level services available for genetic disorders in Iran. For the purpose of this study, essential data were collected from every facility providing community genetic services in Tabriz city of Iran using a prestructured checklist. Technical information was filled in the predesigned forms using diagnostic records of each client/patient. Information was also gathered from community genetic services clients through a face-to-face interview at these facilities to assess the quality of services provided. Primary prevention measures were available in 80 percent of centres in the study population. Diagnostic techniques were fully available in the study area both in public and private sectors. Screening of congenital hypothyroidism and thalassemia has been successfully performed across the country by the Ministry of Health. Other screening programs have also been initiated by the country health authorities for neural tube defects, Down syndrome, and phenylketonuria. The high cost of genetic services at secondary and tertiary levels does not allow many people to get access to these services despite their needs. Governments will therefore need to allocate necessary resources to make the essential genetic services available for everyone needing these in the community.

## 1. Introduction

Prenatal genetic service has a key role in detecting the genetic risks and carrier screening for thehereditary conditions. 

Prenatal genetic service has a key role in detecting the genetic risks and carrier screening for certain hereditary conditions such as sickle cell disease, cystic fibrosis, Tay Sachs disease, and alpha/beta thalassemia. According to the previous studies in the region, the genetic disorders and congenital abnormalities can affect individuals in different ages ranging from 2 percent at birth to 65 percent of individuals in their lifetime period. They account for approximately 30 percent of pediatric hospital admissions ranging from mild to very severe conditions [1, 2]. 

The essential goals of Community Genetic Services (CGS) are to manage the prevention, clinical, and psychosocial demands of individuals suffering from/at risk of hereditary disorders. This can be achieved through timely and precise clinical and laboratory diagnosis, genetic consultation, and primary health care services [3].

As for any disease, prevention methods in genetic disorders are performed at three levels including primary, secondary, and tertiary. Primary prevention is to avoid the occurrence of a disease (i.e., genetic consultation). Secondary prevention methods are to find and treat patients as early as possible (i.e., drug treatments for some heart anomalies). Tertiary prevention includes every effort to reduce the negative effects of the disease through treatment or rehabilitation (i.e., rehabilitation for limb anomalies).

The aim of the study was to report a brief description of the primary, secondary, and tertiary level services available for genetic disorders in Tabriz city of Iran as a sample pattern of CGS for similar regions. 

## 2. Methods

This study was carried out in Tabriz city of Iran. The city is one of the three major cities in the northwest of the country. Health houses, primary care centres, public/private clinics and hospitals, and Welfare and Rehabilitation Organization provide CGS in the area covering about 5 millions of population in the northwest of the Iran. 

For the purpose of this study, essential data were collected from every facility providing CGS in the area using a prestructured checklist. Technical information was filled on the predesigned forms using diagnostic records of each client/patient. Information was also gathered from CGS clients through a face-to-face interview at these facilities to assess the quality of services provided. 

The study took place between March and June 2010. A total number of 19 public and private hospitals and clinics and 15 heath centres were checked for the purpose of this study. A total number of 140 clients/patients were interviewed to assess the quality of services, costs, and so forth. 

Approval for this study was obtained from the Regional Committee of Medical Ethics of Tabriz University of Medical Sciences. Accordingly, an informed consent from the study participants was obtained and data were gathered anonymised at the source of data collection to keep personal information private. 

## 3. Results

Some of the current services at primary, secondary, and tertiary levels available for genetic disorders in Iran are listed in Table 1. 

Primary prevention measures were available in 80 percent of centres and facilities in the study population. These measures include preconceptional care and folic acid supplementation, pregnancy health care services, maternal and children health care services, vaccination (i.e., rubella, measles, etc.), and various educational programmes for the primary prevention of congenital anomalies and genetic disorders. 

Diagnostic techniques including ultrasonography, DNA extract tests (including PCR), serum alpha fetoprotein, nonconjugated estradiol, amniocentesis, chromosomal analysis of amniotic fluid, maternal phenyl alanine test, urine and plasma amino acids analysis fetal echocardiography, MRI scan and CT scan, electroencephalography, chest radiography, electrocardiogram, different type of echocardiography, color ultrasonography, pulse and color Doppler, angiocardiography, pulmonary angiography, liver function tests, IV urography and urethrography, blood gas analysis, chest radiography, radiography by barium swallow, thoracentesis, bronchography, lung function tests, lung biopsy, lung aspiration, bronchoscopy, laryngoscopy, thoracoscopy, radio-nucleotide scan, fluoroscopy, arteriography, and aortography were all fully available in the study area both in public and private sectors.

Screening of congenital hypothyroidism and thalassemia has been successfully performed across the country by the Ministry of Health [4]. Other screening programs have also been planned/started by the country health authorities for neural tube defects, the Down syndrome, and phenylketonuria. 

Selected types of congenital anomalies (i.e., heart defects) were routinely treated by various types of drugs as well as the neurosurgical technique for hydrocephaly and Spina Bifida, shunting for hydrocephaly with or without myelomeningocele, reconstructive surgery for cleft lip, and palate and for patent ductus arteriosus (PDA), ventricular septal defect (VSD), Atrioventricular (AV) canal, tetralogy of fallot, pulmonary valve stenosis and noncyanotic congenital heart diseases, urology corrective surgery for hypospadias, cryptorchidism, undiscerning testis, arterioplasty for coarctation aorta, valvuoplasty and valve replacement for aortic valve stenosis, craniosynostosis surgery, dialysis and kidney transplants for congenital renal diseases, and bone marrow transplantation for different type of bone marrow failure. 

## 4. Discussion

In this study, we reported the current status of genetic services for the prevention, treatment, and rehabilitation of congenital anomalies in Iran provided by public and private sectors in the area.

This study showed that primary prevention of congenital anomalies and genetic disorders (i.e., folic acid supplementation before and during pregnancy, etc.) is now well established in the region. Despite some differences, similar findings have been reported in previous studies [5–7] indicating that the activities by the Ministry of Health and private sectors for the prevention of genetic disorders are well understood/accepted in the country. Research reports from the nine European countries, Georgia, Venezuela, and India showed that primary prevention measures have been implemented in these regions too [8–11]. However, the structure of the preventive strategies may vary by the region based on the population demands and financial sources. 

Our study indicated that despite availability of diagnostic and treatment facilities for genetic disorders in the area, they are mostly too expensive to be fully accessible for many people in the country as they responded in the face-to-face interview. It is obvious that as more advances in genetic technology occurring, the demands for CGS are growing in the population. However, the high cost of genetic services does not allow many people to get access to these services despite their needs. Governments will therefore need to allocate necessary resources to make the essential genetic services available for everyone needing that in the community. More investigations are needed to assess the cost effectiveness and health economics aspects of this matter.

## Figures and Tables

**Table 1:** Community Genetic Services in Iran.

Services	Congenital abnormalities
Alpha fetoprotein	Neural tube defects, nephrosis, chromosomal abnormalities, omphalocele
Amniocentesis	Neural tube defects, chromosomal abnormalities
Amniotic fluid chromosomal analysis	Trisomy 13, 18, 21 and chromosomal abnormalities
Aortography	Congenital heart abnormalities
Arteriography	Congenital heart abnormalities
Arterioplasty with balloon	Coarctation aorta
Blood gas analysis	Congenital respiratory diseases
Bone marrow transplantation	Hydroxyurea
Bronchography	Congenital respiratory diseases
Bronchoscopy	Congenital respiratory diseases
Chest radiography	Congenital respiratory diseases
Chest radiography	Congenital heart abnormalities
Chorionic gonadotropin level	Trisomy 13, 18, 21
Color ultrasonography and ventriculography	Congenital heart abnormalities
Cordocentesis services	Hemoglobinopathies, fetal anemia, acid-base disorders, thalassemia
Craniosynostosis surgery	Nervous system anomalies
Dialysis and kidney transplants	Congenital renal diseases
DNA services	Thalassemia, muscular dystrophy, phenylketonuria (PKU), Turner syndrome, cystic fibrosis
Drug treatments	Congenital heart abnormalities
Echoencephalography procedure	Neural tube defects
Electrocardiogram	Congenital heart abnormalities
Embryo echocardiography	Congenital heart abnormalities
Embryoscopy	Facial and limb abnormalities
Exercise testing, congenital heart disease	Congenital heart abnormalities
Fetal skin biopsy	Albinism
Fetoscopy	Limb abnormalities
Fluoroscopy	Congenital heart abnormalities
Genital ambiguous surgery	Urogenital malformations
Growth monitoring	Congenital anomalies diagnosed after birth
Hemoglobin electrophoresis	Sickle cell anemia
Herpes and cytomegalovirus tests	Mother and fetus
Intrauterine surgery	Diaphragmatic hernia and myelomeningocele
Iron levels and hemoglobin electrophoresis	Thalassemia
IV urography and urethrography	Obstructive urethral congenital disease
Laryngoscopy	Congenital respiratory diseases
Liver function tests	Hemochromatosis
Lung aspiration	Congenital respiratory diseases
Lung biopsy	Congenital respiratory diseases
Lung function tests	Congenital respiratory diseases
Maternal health care services	Congenital anomalies
Maternal phenyl alanine	Fetal microcephaly
MRI scan and CT scan	Neural tube defects (hydrocephaly and spina bifida)
Neurosurgical techniques	Hydrocephaly and Spina Bifida
NICU and special delivery services	Pregnancies with omphalocele
Nonconjugated acetyl test	Trisomy 21
Preconceptional folic acid consumption	Congenital anomalies
Pregnancy health care services	Congenital anomalies
Public education	Congenital anomalies
Pulse and color doppler, angiocardiography	Congenital heart abnormalities
Radiography by barium swallow	Congenital respiratory diseases
Radio-nucleotide scan	Congenital respiratory diseases
Reconstructive surgery	VSD, PDA, AV, tetralogy of fallot, pulmonary valve stenosis, and noncyanotic congenital heart diseases
Reconstructive surgery	Cleft lip and palate
Screening programme	Congenital hypothyroidism, adrenal hyperplasia
Shunting	Myelomeningocele with hydrocephaly
Surgical treatment	Neurofibromatosis
Thoracentesis	Congenital respiratory diseases
Thoracoscopy	Congenital respiratory diseases
Treatment by dexamethasone	High risk pregnancy for congenital adrenal hyperplasia
Two-dimensional echocardiography	Congenital heart abnormalities
Ultrasound diagnostic services	Neural tube defects, heart defects, diaphragmatic hernia
Urine and plasma amino acids, serum ammonium	Fetal microcephaly
Urology corrective surgery	Hypospadias, cryptorchidism, undescending testis
Vaccination	Congenital defects caused by viral infections
Valvuloplasty and valve replacement	Aortic valve stenosis
Varios clinical tests	Eye defects, thyroid anomalies, speech and hearing defects
